# Effects of two genes coding squamous cell carcinoma antigen on the diagnosis and treatment of cervical squamous cell carcinoma

**DOI:** 10.12669/pjms.302.4374

**Published:** 2014

**Authors:** Qi’nan Yin, Ying Wang, Chenchen Zheng, Yanjuan Liu, Zhuo Chen, Fuer Lu, Guangying Huang

**Affiliations:** 1 Qi’nan Yin, Institute of Integrated Traditional Chinese & Western Medicine, The Affiliated Tongji Hospital of Tongji Medical College, Huazhong University of Science & Technology, Wuhan 430030, P. R. China.; 2 Ying Wang, Institute of Integrated Traditional Chinese & Western Medicine, The Affiliated Tongji Hospital of Tongji Medical College, Huazhong University of Science & Technology, Wuhan 430030, P. R. China.; 3 Chenchen Zheng, Institute of Integrated Traditional Chinese & Western Medicine, The Affiliated Tongji Hospital of Tongji Medical College, Huazhong University of Science & Technology, Wuhan 430030, P. R. China.; 4 Yanjuan Liu, Institute of Integrated Traditional Chinese & Western Medicine, The Affiliated Tongji Hospital of Tongji Medical College, Huazhong University of Science & Technology, Wuhan 430030, P. R. China.; 5 Zhuo Chen, Institute of Integrated Traditional Chinese & Western Medicine, The Affiliated Tongji Hospital of Tongji Medical College, Huazhong University of Science & Technology, Wuhan 430030, P. R. China.; 6 Fuer Lu, Institute of Integrated Traditional Chinese & Western Medicine, The Affiliated Tongji Hospital of Tongji Medical College, Huazhong University of Science & Technology, Wuhan 430030, P. R. China.; 7 Guangying Huang, Institute of Integrated Traditional Chinese & Western Medicine, The Affiliated Tongji Hospital of Tongji Medical College, Huazhong University of Science & Technology, Wuhan 430030, P. R. China.

**Keywords:** Cervical squamous cell carcinoma, Squamous cell carcinoma antigen, Tumor marker

## Abstract

***Objective: ***To study the effects of expressions of SCCA1 and SCCA2 in cervical squamous cell carcinoma on its diagnosis, treatment evaluation and prognosis analysis.

***M*** ***ethod*** ***s*** ***:*** Seventy-six cervical squamous cell carcinoma patients enrolled in our hospital from October 2011 to April 2013 were selected, and another 76 healthy females (without cervical tissue lesions) were enrolled as the control. SCCA1 and SCCA2 expressions in the two groups were compared by RT-PCR. The serodiagnosis results before and after chemotherapy were compared to clarify the effects of SCCA2 expression.

***Results:*** The two groups had similar relative SCCA1 expression rates that were not significantly correlated with pathological factors. Before chemotherapy, the relative expression rates of SCCA2 were significantly higher in the patients with later stage (t=6.018, P=0.00082<0.05) and lymphatic metastasis (t=6.281, P=0.00192<0.05). After treatment, relative SCCA2 expression rate was decreased more significantly in the effective group than that in the ineffective group (t=10.27893, P=0.02815<0.05).

***Conclusion: ***The expression of SCCA1 failed to indicate the onset, diagnosis and prevention of cervical squamous cell carcinoma, whereas that of SCCA2 worked as one of the tumor markers.

## INTRODUCTION

Currently, tumor markers provide clues for the clinical diagnosis, prevention and treatment of several cancer patients.^1^ For women, cervical cancer is one of the malignant tumors threatening health. According to the American Cancer Society, more than 12,200 American women were diagnosed as invasive cervical cancer in 2010, of which squamous cell carcinoma was most common. Chinese cervical cancer patients account for 1/3 of the patients worldwide, the number of which is now rapidly rising.^2^ Squamous cell carcinoma antigen (SCCA), as the serine protease inhibitor (Serpin) family of proteins, can be used as a biomarker for aggressive squamous cell carcinoma in cancers of cervix, lung and liver in case of elevated expression. Recently, detecting SCCA is crucial in the diagnosis of cervical squamous cell carcinoma.^3^ For instance, Yan et al.^4^ reported that expressions of SCCA1 mRNA and SCCA2 mRNA functioned differently in diagnosing tumors.

In this study, the expressions of SCCA1 mRNA and SCCA2 mRNA in 76 cervical squamous cell carcinoma patients enrolled in our hospital were detected, aiming to explore their influences on the diagnosis, treatment evaluation and prognosis analysis of cervical squamous cell carcinoma.

## METHODS

***Clinical Data: ***Seventy-six cervical squamous cell carcinoma patients enrolled in our hospital from October 2011 to April 2013 were included in this study. They were aged 28-67 years old, with the mean age of 55.2 years old. They were then classified into 35 cases of T1-T2 stages, and 41 cases of T3-T4 stages according to TNM staging criteria. According to clinical staging criteria, they were classified into 32 cases of Stage I, 25 cases of Stage II, and 19 cases of Stage III. Of all patients, 23 had mothers who once suffered from cervical squamous cell carcinoma, and the other 53 did not have family history. Fresh tissues of surgically resected tumors were sampled (biopsy). Meanwhile, cervical tissues of 76 normal women were sampled as the control group.

***Experimental Concept: ***Generally, serum SCCA2 content was tested for overall SCCA analysis. Since both SCCA1 and SCCA2 mRNAs were detected in this study, enzyme-linked immunosorbent assay (ELISA) was not applicable as the first step. Therefore, the relative expression ratio of two genes was measured by RT-PCR:^4^ △Ct=△Ct_target gene_-△Ct_internal reference gene_. Meanwhile, sera were sampled before and after chemotherapy, and SCCA2 expression changes were determined by ELISA.

***Materials and*** ***A*** ***pparatus: ***We herein used diethyl pyrocarbonate (DEPC)-treated water, agarose (Shang Yisha Biotechnology Co., Ltd.), TRizol reagent (Invitrogen, USA), M-MLV reverse transcriptase (Tianjin Bomeike Biotechnology Co., Ltd.), RNase-free water (Shanghai Shifeng Biotechnology Co., Ltd.), agarose gel electrophoresis system (Shenzhen Cy-Tech Biotechnology Co., Ltd.), LightCycler 480 II real-time fluorescence quantitative PCR analyzer (Shanghai Hengjiu Medical Instruments Co., Ltd.), and SCCA ELISA kit (Shang Yisha Biotechnology Co., Ltd.).

**Fig.1:**
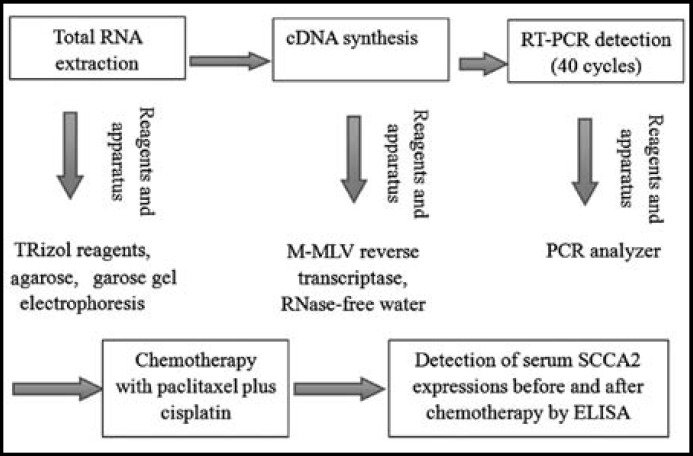
Detailed experimental procedure

**Experimental** **P** **rocedure: **The detailed experimental procedure is schematized in Fig.1.

**Detailed** **P** **rocedure** ^5^^-^^8^

***Experiments before treatment: ***Cervical tissues of the two groups were subjected to RNA extraction by TRizol reagent, and cDNA was synthesized by M-MLV, with the product stored at -80°C. Reference gene: GAPDH, upstream primer sequence: 5'-CCA CCC ATG GCA AAT TCC ATG-3'; downstream primer sequence: 5'-TCT AGA CGG CAG GTC AGG TCC-3'. SCCA1: upstream primer sequence: 5'-TGA ATT CAC TCA GTG AAG CTA-3'; downstream primer sequence: 5'-AAT CAA AGA AGG GAC AAC ATC-3'. SCCA2: upstream primer sequence: 5'-TTG CAC TTG ATC CTG TTC CAA-3', downstream primer sequence: 5'-CGT ACT TAG GGG GCC CAA GGA-3'. Then PCR amplification was performed.

***Experiments after treatment: ***Sera were collected before treatment. Patients were subjected to chemotherapy with paclitaxel combined with cisplatin for three weeks. They were first infused with 150 mg/m^2^ paclitaxel and then 60 mg/m^2^ cisplatin three hours later. Peripheral bloods were collected, and the SCCA2 contents were detected by SCCA2 ELISA kit, with the values >1.5 mg/L being abnormally expressed.

***Statistical*** ***A*** ***nalysis: ***All data were analyzed by SPSS 15.0 and expressed as x±s. The measurement data were compared with t test. P<0.05 was considered statistically significant.

## RESULTS

**Fig.2:**
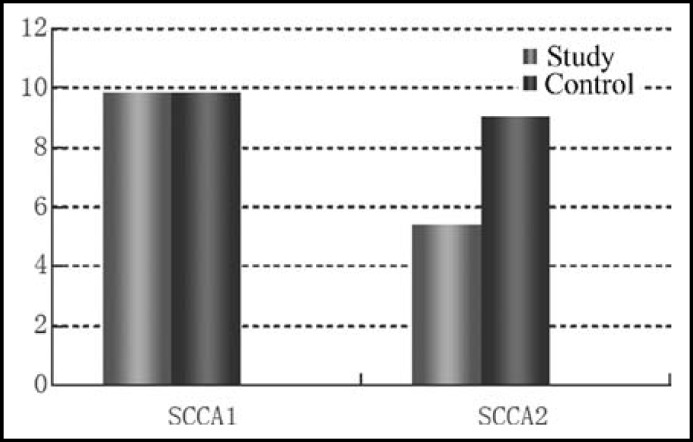
Relative expression rates of SCCA1 and SCCA2 before treatment

***Relative*** ***E*** ***xpression*** ***R*** ***ates of SCCA1 and SCCA2 before*** ***T*** ***reatment: ***The two groups had similar relative SCCA1 expression rates (P>0.05), whereas the relative SCCA2 expression rate of the study group (4.396±2.002) was significantly (P=0.0000982<0.05) lower than that of the control group (9.028±3.182) (Table-I and Fig.2).

**Fig.3:**
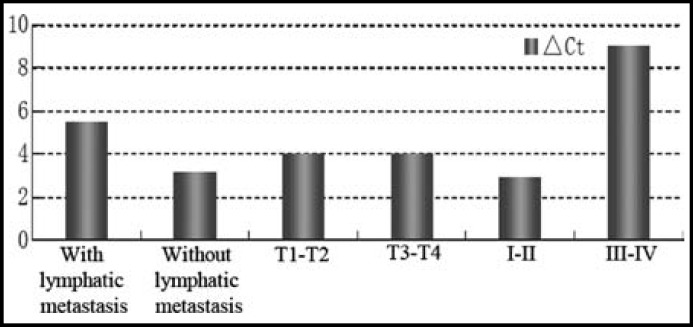
Relative SCCA2 expression rates

***Relative SCCA2 Expression Rate and Correlated Factors: ***Relative SCCA2 expression was significantly correlated with lymphatic metastasis, T classification and clinical stage. Relative SCCA2 expression rates were higher in the patients with lymphatic metastasis (t=6.281, P=0.00192<0.05) and later clinical stages (t=6.018, P=0.00082<0.05). However, the rates did not differ with varied age, T classification and family history (P>0.05) (Table-II, Fig.3).

***SCCA2*** ***E*** ***xpressions after*** ***T*** ***reatment: ***Fifty-three patients were treated effectively by chemotherapy, while the 23 patients were not. By using ELISA, we found that the effective group ((6.93±2.89) mg/L) experienced more significantly (t=10.27893, P=0.02815<0.05) decreased average SCCA2 expression rate than the ineffective group did ((1.56±1.68) mg/L) (Table-III).

## DISCUSSION

Cervical cancer mainly threatens women, and those in China (over 40 thousands) accounted for 1/3 of the patients worldwide. As the milestone of cervical cancer studies, Kato and Torigoe^9^ successfully extracted SCCA from cervical squamous cell carcinoma in 1977. Thus, investigating SCCA expression is key in diagnosing and preventing this type of cancer.

**Fig.4:**
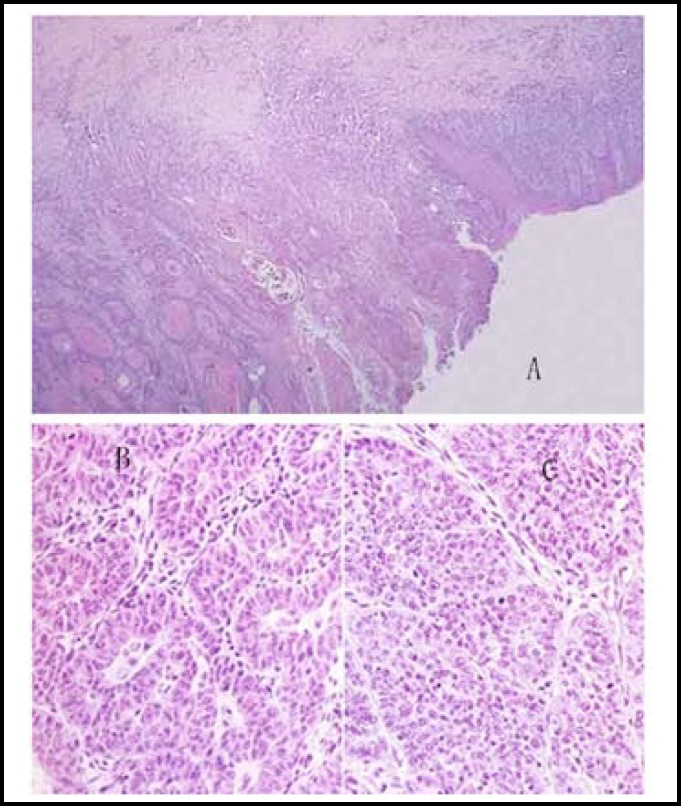
Staining sections of SCCA1 expression under (A) low magnification microscope, (B) medium magnification microscope 1, and (C) medium magnification microscope 2

**Fig.5:**
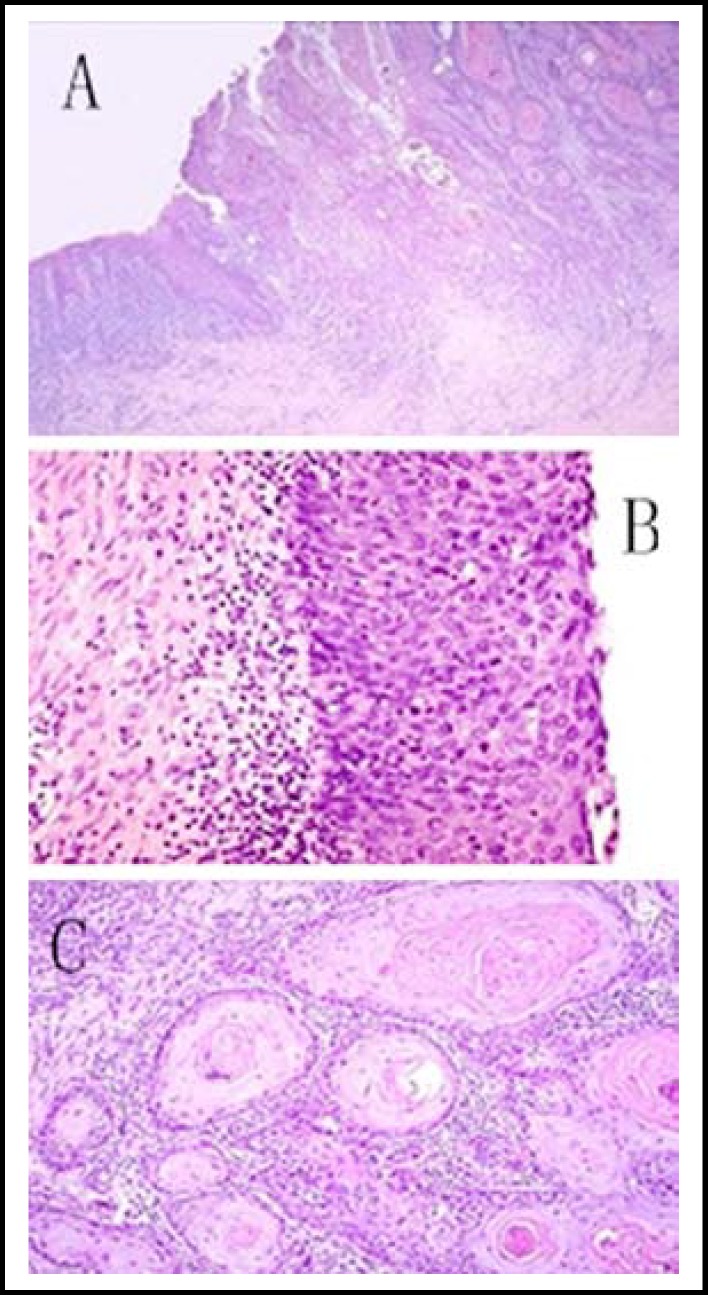
Tissue sections of cervical squamous cell carcinoma under (A) low magnification microscope, (B) medium magnification microscope, and (C) high magnification microscope

**Table-I:** Relative expression rates of SCCA1 and SCCA2 before treatment

	*n*	*SCCA1*	*SCCA2*
Study	76	9.827±1.291	5.396±2.002
Control	76	9.801±1.012	9.028±3.182
t		-0.0142	7.9210
P		>0.05	<0.05

**Table-II:** Relative SCCA2 expression rates

*Factor*		*n*	*△* *Ct*	*t*	*P*
Age^1^	28-55	31	5.021±2.003	-1.435	0.78514
	55-67	45	5.192±2.157		
Lymphatic metastasis^2^	Yes	40	5.552±3.021	6.281	0.00192
	No	36	3.192±2.018		
T classification^3^	T1-T2	35	4.023±3.012	-1.321	0.35475
	T3-T4	41	4.021±3.014		
Clinical staging^4^	I-II	57	2.901±1.023	6.018	0.00082
	III-IV	19	9.017±2.032		
Family history^5^	Yes	23	5.281±1.821	1.28194	0.098125
	No	53	5.301±1.390		

P_1_, P_3_>0.05; P_2_, P_4_<0.05

**Table-III:** SCCA2 expression changes before and after chemotherapy

	*n*	*Decreasing amplitude*	*t*	*P*
Effective	53	6.93±2.89	10.27893	0.02815
Ineffective	23	1.56±1.68		

In this study, relative SCCA1 expression rates of the two groups were similar (P>0.05), suggesting that SCCA1 expression did not exert specific effects on the onset and diagnosis of cervical cancer. In contrast, relative SCCA2 expression rate can be used as a specific tumor marker because it was higher in the patients with lymphatic metastasis, as was lower in those with later clinical stage. Besides, we also verified the patients with significantly decreased SCCA2 expressions as the effective treatment group (earlier clinical stage) after chemotherapy.

SCCA that was positively expressed in tumor tissues was stained grayish brown (or reddish brown). Although this method directly indicates the degree of SCCA expression, it is only applicable in qualitative analysis. The specimens are shown below (Fig. 4 and 5).^10^

Besides being the marker of cervical squamous cell carcinoma, SCCA is also the marker of lung cancer.^11^^-^^13^ SCCA1 and SCCA2 are the genes coding SCCA that is intrinsically a serine protease inhibitor, and SCCA2 is located next to the gene breakpoint of 18-trisomysyndrome patients.^14^ The two genes, which share a homology of 92%, code neutral and acid morphogenetic proteins respectively.^15^^,^^16^ Given that most SCCA-related studies only focused on overall SCCA genes, detecting SCCA2 may work in evaluating therapeutic effects.^17^ In other words, relative SCCA2 expression rate is bound to reduce after treatment, accompanied by decreased clinical stage. The two-step verification analysis in this study accurately and directly confirmed that detecting SCCA2 expression is informative.

It has previously been reported that SCCA was able to regulate the expression of antigen-E protein associated with cell migration and invasion.^18^ As this protein is a prognostic factor related with the invasion and metastasis of cervical squamous cell carcinoma, SCCA can be used to ensure lymphatic metastasis occurs. SCCA2 was used instead of SCCA in practice because SCCA1 and SCCA2 function differently. In treatment and prevention, SCCA content changes have long been used to sensitively indicate drug administration, radiotherapy, chemotherapy and aggravated symptoms. Therefore, SCCA content plays an essential role in real-time diagnosis of cervical squamous cell carcinoma.

In recent years, tumor markers, such as SCCA, have been spotlighted in the diagnosis of cervical cancer. However, in addition to SCCA, serum CYFRA21-1 and CEA are also the markers of lung cancer. In the meantime, Xiang^19^ reported that CAl25, CAl5.3, SCCA and ck20 mRNA were positively expressed in female pelvic tumors, and they were negatively expressed in benign ones or in normal people, suggesting that the markers were actually correlated with diagnosis and clinical stage. For treatments on the molecular level, tumor markers are focused, the genes of which are commonly utilized to treat specific diseases for further improvements.

However, studies on SCCA1 and SCCA2 remain limited, especially for the debate on the correlation between SCCA and pathological parameters of cervical squamous cell carcinoma. Hence, it is imperative to conduct more large-sample-size clinical studies. The detailed functions of “SCCA1 promoter” in genetic treatment are still undefined. In the meantime, the positive rates of SCCA may differ significantly depending on the patients themselves and detection protocols. As a result, clarifying the effects of SCCA1 and SCCA2 on clinical detection and prognosis is particularly necessary, which requires top-notch detection techniques and large-scale studies.

In summary, we found that only SCCA2 was expressed significantly differently in cervical squamous cell carcinoma patients and normal controls. The patients with lymphatic metastasis had higher relative SCCA2 expression rates, thus allowing SCCA2 to be an eligible marker for the determination of lymphatic metastasis and prognosis.

## Authors’ Contribution:

**Qi’nan Yin** **,** **Guangying Huang** **:** Study concept and design. **Qi’nan Yin, Ying Wang, Chenchen Zheng, Yanjuan Liu, Zhuo Chen, Fuer Lu, Guangying Huang:** Analysis and interpretation of data. **Qi’nan Yin, Guangying Huang:** Critical revision of the manuscript for important intellectual content. **Qi’nan Yin:** Statistical analysis.

## References

[1] Sheng X, Du X, Zhang X, Li D, Lu C, Li Q (2009). Clinical value of serum HMGB1 levels in early detection of recurrent squamous cell carcinoma of uterine cervix: comparison with serum SCCA, CYFRA21-1, and CEA levels. Croat Med J.

[2] Luan XM, Wang SZ, Zhang SL (2012). Progress of squamous cell carcinoma antigen in cervical squamous cell carcinoma. J Int Obstec Gynecol.

[3] Nakamura K, Okumura Y, Kodama J, Hongo A, Kanazawa S, Hiramatsu Y (2010). The predictive value of measurement of SUVmax and SCC-antigen in patients with pretreatment of primary squamous cell carcinoma of cervix. Gynecol Oncol.

[4] Yan LJ, Zhao X, Shao SL (2011). Expression of SCCA1 and SCCA2 in cervical squamous cell carcinoma and its clinical significance. J Shanxi Med Univ.

[5] Catanzaro JM, Guerriero JL, Liu J, Ullman E, Sheshadri N, Chen JJ (2011). Elevated expression of squamous cell carcinoma antigen (SCCA) is associated with human breast carcinoma. PloS one.

[6] Olsen JR, Dehdashti F, Siegel BA, Zighelboim I, Grigsby PW, Schwarz JK (2011). Prognostic utility of squamous cell carcinoma antigen in carcinoma of the cervix: association with pre- and posttreatment FDG-PET. Int J Radiat Oncol Biol Phys.

[7] Biasiolo A, Tono N, Zaninotto M, Merkel C, Fassina G, Plebani M (2013). Specificity of squamous cell carcinoma antigen (SCCA)-IgM detection in patients with HCV infection and rheumatoid factor seropositivity. J Med Virol.

[8] Patel JL, Erickson JA, Roberts WL, Grenache DG (2010). Performance characteristics of an automated assay for the quantitation of CYFRA 21-1 in human serum. Clin Biochem.

[9] Kato H, Torigoe T (1977). Radioimmunoassay for tumor antigen of human cervical squamous cell carcinoma. Cancer.

[10] Kantyka T, Potempa J (2011). Human SCCA serpins inhibit staphylococcal cysteine proteases by forming classic "serpin-like" covalent complexes. Methods Enzymol.

[11] Marin-Muller C, Li M, Chen C, Yao Q (2009). Current understanding and potential immunotherapy for HIV-associated squamous cell carcinoma of the anus (SCCA). World J Surg.

[12] Van't Westeinde SC, van Klaveren RJ (2011). Screening and early detection of lung cancer. Cancer J.

[13] Choi MK, Park YH, Hong JY, Park HC, Ahn YC, Kim K (2010). Clinical implications of esophagorespiratory fistulae in patients with esophageal squamous cell carcinoma (SCCA). Med Oncol.

[14] Suzuki M, Deng Z, Hasegawa M, Uehara T, Kiyuna A, Maeda H (2012). Squamous cell carcinoma antigen production in nasal inverted papilloma. Am J Rhinol Allergy.

[15] Trerotoli P, Fransvea E, Angelotti U, Antonaci G, Lupo L, Mazzocca A (2009). Tissue expression of Squamous Cellular Carcinoma Antigen (SCCA) is inversely correlated to tumor size in HCC. Mol Cancer.

[16] Beale G, Chattopadhyay D, Gray J, Stewart S, Hudson M, Day C (2008). AFP, PIVKAII, GP3, SCCA-1 and follisatin as surveillance biomarkers for hepatocellular cancer in non-alcoholic and alcoholic fatty liver disease. BMC Cancer.

[17] Giannini EG, Basso M, Bazzica M, Contini P, Marenco S, Savarino V (2010). Successful antiviral therapy determines a significant decrease in squamous cell carcinoma antigen-associated (SCCA) variants' serum levels in anti-HCV positive cirrhotic patients. J Viral Hepatitis.

[18] Yin M, Hou Y, Zhang T, Cui C, Zhou X, Sun F (2013). Evaluation of chemotherapy response with serum squamous cell carcinoma antigen level in cervical cancer patients: a prospective cohort study. PLoS One.

[19] Xiang W (2012). Clinical significance of CA125, CA15-3, SCCA and ck20 mRNA in detection of gynecological pelvic tumors. Chin J Gerontol.

